# PFC@O_2_ Targets HIF-1α to Reverse the Immunosuppressive TME in OSCC

**DOI:** 10.3390/jcm12020560

**Published:** 2023-01-10

**Authors:** Zhou Lan, Ke-Long Zou, Hao Cui, Hao Chen, Yu-Yue Zhao, Guang-Tao Yu

**Affiliations:** Stomatological Hospital, Southern Medical University, Guangzhou 510280, China

**Keywords:** oral squamous cell carcinoma, hypoxia, HIF-1α, perfluorocarbons, tumor microenvironment

## Abstract

As a typical hallmark of solid tumors, hypoxia affects the effects of tumor radiotherapy, chemotherapy, and photodynamic therapy. Therefore, targeting the hypoxic tumor microenvironment (TME) is a promising treatment strategy for cancer therapy. Here, we prepared an Albumin Human Serum (HSA)-coated perfluorocarbon (PFC) carrying oxygen (PFC@O_2_) to minimize OSCC hypoxia. The results showed that PFC@O_2_ significantly downregulated the expression of HIF-1α and the number of M2-like macrophages in vitro. Furthermore, PFC@O_2_ effectively inhibited the growth of oral squamous cell carcinoma (OSCC) and reduced the proportion of negative immunoregulatory cells, including myeloid-derived suppressor cells (MDSCs) and M2-like macrophages of TME in a 4-nitroquinoline N-oxide (4NQO)-induced mouse model. Conversely, the infiltration of CD4^+^ and CD8^+^ T cells was significantly increased in TME, suggesting that the anti-tumor immune response was enhanced. However, we also found that hypoxia-relative genes expression was positively correlated with CD68^+^/CD163^+^ TAMs in human tissue specimens. In summary, PFC@O_2_ could effectively inhibit the progression of OSCC by alleviating hypoxia, which provides a practical basis for gas therapy and gas synergistic therapy for OSCC.

## 1. Introduction

Cancer of the lip and oral cavity, which ranks eighteen among malignancies, is still a significant public health concern [1]. The vast majority of them arise from the oral epithelium, and 90% belong to oral squamous cell carcinoma (OSCC) [2]. The multimodal treatment methods for OSCC include surgical resection, adjuvant radiotherapy and chemotherapy, immunotherapy, targeted therapy, etc. [3]. However, the five year survival rate is low at 60% [4]. The major obstacle to treatment is clinical therapy resistance, which may be attributable to hypoxia, a typical hallmark of the tumor microenvironment (TME) in almost all solid tumors [5].

Tumor hypoxia is mainly caused by an imbalance between the low oxygen supply caused by abnormal vascularization and the high oxygen consumption of rapidly proliferating tumor cells [6]. Hypoxia Inducible Factor-1α (HIF-1α) is a transcription factor that is overexpressed in a variety of solid tumors under hypoxic conditions, and it is often associated with a poor prognosis [7]. In normoxia, HIF-1α could be degraded via an ubiquitin-proteasome pathway within five minutes, which includes hydroxylation of proline residues and acetylation of a lysine residue [8,9]. While under hypoxia, HIF-1α will not be degraded and thus form HIF-1 to activate a panel of target genes, e.g., GLUT1 and CA IX [10]. HIF-1α has also been identified as the core factor of the hypoxia signaling pathway, which is involved in tumor angiogenesis, proliferation, invasion, metastasis, and even tumor immune suppression [5,11,12].

Recent studies found that hypoxia was closely related to the formation of a tumor immunosuppressive microenvironment (TIME) [5,6,13]. Among TIME, MDSCs and tumor associated macrophages (TAMs) play an important role in tumor immune suppression [14,15]. Jung et al. demonstrated that hypoxia-induced tumor exosomes can affect the recruitment and polarization of macrophages to achieve immune escape [16]. David et al. revealed that HIF-1α could account for the accumulation of MDSCs in hepatocellular carcinoma (HCC) via chemokines (C-C motif ligand 26 (CCL26)) and maintenance by Ectonucleoside Triphosphate Diphosphohydrolase 2 (ENTPD2), which is a direct transcriptional target of HIF-1α [17]. In malignant melanoma, targeted inhibition of HIF-1α induced high levels of CCL2 and CCL5 expression and then increased the invasion of tumor-killing NK cells, CD3^+^, CD4^+^, and CD8^+^ T cells in tumor tissues [18].

Given the close relationship between hypoxia and tumor development and treatment, improving hypoxic TME may be a promising therapeutic strategy [19]. Since 1966, perfluorocarbons (PFC) have attracted extensive attention from researchers due to their superior oxygen storage capacity and have been used as a substitute for red blood cells in artificial blood [20,21]. Its nanoscale size enables it to transport oxygen to the smallest capillaries, directly reach the TME, and ameliorate hypoxia [22]. Recent studies have shown that PFC can carry oxygen to produce a large number of reactive oxygen species to overcome hypoxia treatment resistance and enhance the efficacy of tumor radiotherapy, immunotherapy, and photodynamic therapy [23,24,25].

As a result, we speculate that PFC may serve as an excellent oxygen carrier to achieve a hypoxic tumor site to exert supposing tumor inhibition. In our experiment, HSA was used to encapsulate PFC to increase its water solubility, and then the oxygen loading capacity was first measured. For an in vitro study, we tested whether PFC@O_2_ could be taken into SCC7 cells and accelerate the degradation of HIF-1α by confocal and Western blotting. We also examined the suppressing capacity of PFC@O_2_ to inhibit M2-like macrophage polarization. A 4-nitroquinoline N-oxide (4NQO)-induced mouse model was used to elucidate the effective inhibiting ability of PFC@O_2_ in OSCC by IHC and flow cytometry analysis. Finally, the clinical data revealed the correlation between HIF-1α and CD68^+^/CD163^+^ M2 TAM to clarify the potential for a PFC@O_2_ treatment strategy.

## 2. Materials and Methods

### 2.1. Clinical Sample Collection

Clinical samples (*n* = 55) were collected after obtaining the approval of the Medical Ethics Committee of the Stomatological Hospital affiliated with Southern Medical University and the informed consent of all patients. Freshly collected samples were fixed with 4% paraformaldehyde, dehydrated with gradient alcohol, and then directly embedded in paraffin for use.

### 2.2. Synthesis of PFC@O_2_

A total of 600 μL of PFC (TCI, CAS RN: 97571-69-2, I1043) were added to 1.4 mL of PBS containing 14 mg of HSA (Solarbio, A8230, China). After ultrasonication (100 W, 200 s) with an ice bath, the samples were centrifuged at 8000 rpm for 3 min, resuspended in 1 mL of PBS, and passed through O_2_ (at a flow rate of 5 L/min for 1 min) for use.

### 2.3. Oxygen Storage Capacity

A total of 1 mL of PFC@O_2_ was diluted 25-fold with PBS. Water was used as the control group. Subsequently, the PFC@O_2_ PBS solution was magnetically stirred at 600 rpm, and the oxygen content in the solution was detected by a portable oxygen meter. Data were collected every 30 seconds after the oxygen-carrying meter reading was stable.

### 2.4. Cell Lines

SCC7 and RAW 264.7 murine macrophages were, respectively, purchased from the Guangzhou yuan jing Biotechnology Co., Ltd. (China), and the American Type Culture Collection (ATCC, Manassas, VA, USA). A STR sequence was used for genotype confirmation. Both were cultured in Dulbecco’s Modified Eagle Medium (DMEM), a 10% fetal bovine serum (FBS), at 5% CO_2_ and a temperature of 37 °C in a humidified incubator according to relative guidelines.

### 2.5. Western Blot

A total of 1 × 10^6^ SCC7 were inoculated into a 10 cm dish and precultured for 12 h. Three groups were set up: normoxia (21% O_2_) without treatment and hypoxia (5% O_2_) with or without PFC@O_2_ 50 uL treatment for 24 h. Each group was repeated three times. An anti-HIF-1α antibody was purchased from Chengdu Zen Biotechnology Co., Ltd. (340462, 1:1000, China). An anti-β-actin antibody was purchased from Proteintech (66009-1-Ig, 1:1000).

### 2.6. Detection of M2-Like Macrophage Polarization

A total of 3 × 10^5^ RAW264.7 cells and 3 × 10^5^ SCC7 were co-cultured in 6-well plates for 12 h. Three groups were set up: group 1, normoxia; group 2, hypoxia without treatment; and group 3, hypoxia with 50 μL of PFC@O_2_. Flow cytometry was used to determine the ratio of CD206^+^ M2-like macrophages after 24 hours of incubation at 37 °C.

### 2.7. In Vivo Study of 4NQO-Induced OSCC In Situ

Twelve C57BL/6j mice (6–8 weeks old) were used to induce orthotopic OSCC. A total of 50 μg/mL of 4NQO (Sigma, N8141-5G, USA) was added to the drinking water for mice, and the drinking water was changed weekly. After 4 months, the mice were fed with purified water. Ten tumor-bearing mice were randomly divided into two groups. The experimental group was given i.v. PFC@O_2_ 8 mL/kg per mouse twice a week for two weeks. At the end of the experiment, the mice were euthanized. The tongue, spleen, and lymph nodes were dissected for subsequent detection. The animal experiments were approved by the Animal Ethics Committee of Southern Medical University. All experimental procedures were conducted in accordance with the ARRIVE 2.0 Guidelines.

### 2.8. Flow Cytometry Analysis

Flow cytometry was used to detect the immune cell population in tumor tissues, spleen, and lymph nodes of mice in vivo and the ratio of CD206^+^ M2-like macrophages in vitro. Sample processing was referred to in the article [26]. The CD45^+^ immune cells were enriched using the CD45 Positive Selection Kit (STEMCELL, 18945) according to the instructions before staining. Flow antibodies that we used refer to a previous article [27].

### 2.9. Hematoxylin and Eosin (HE) Staining and Immunohistochemical (IHC) Staining Analysis

The kit for HE staining was purchased from AiBIxin Biotechnology Co., Ltd. (abs9217, China), the citrate antigen retrieval solution for IHC was purchased from Xinheng Biological Technology Co., Ltd. (Biosharp, BL619A, China), the anti-rabbit and anti-mouse secondary antibody kit was purchased from Beijing Zhongshan Jinqiao Biotechnology Co., Ltd. (PV-6001, China), the primary antibody PCNA (CST, 13110T, 1:10000, USA) and the HIF-1α was purchased from Chengdu Zen Biotechnology Co., Ltd. (340462, 1:200), and the primary CD163 polyclonal antibody (16646-1-AP, 1:4000) and CA IX monoclonal antibody (66243-1-Ig, 1:400) were purchased from Wuhan Sanying Biotechnology Co., Ltd(China). A DAB chromogenic kit was purchased from Fuzhou Maixin Biotechnology Development Co., Ltd. (DAB-0031, China). All paraffin sections and the stain procedure refer to our previous study [28].

### 2.10. Cell Intake

Cy5-PEG2000-NH_2_ (Ruixibo^TM^, R-1019-2k) was used to label PFC@O_2_. Briefly, 10 mg of Cy5-PEG2000-NH_2_ was added into 1 mL of PFC@O_2_ solution. The solution was sonicated in an ice bath for 30 min and then stirred in the dark for 6 h. Finally, the solution mixture was centrifuged at 8000 rpm for 3 min and washed twice with PBS to remove the excess Cy5-PEG2000-NH_2_. A total of 1 × 10^5^ SCC7 cells were seeded into 12-well plate slides and precultured for 12 h, followed by co-incubation for 6 h with or without Cy5-labeled PFC@O_2_. After discarding the culture medium and washing twice with PBS, the cells were fixed with 4% paraformaldehyde for 10 min. Subsequently, SCC7 cells were stained sequentially with Neuro-DiO (BIORIGN, BN14021, China) and Hoechst 33342 (biosharp, BL803A) for membrane and nucleus staining according to the instructions.

### 2.11. Data Analysis

Data analyses were performed with GraphPad Prism version 8.0 for Windows (GraphPad Software Inc., La Jolla, CA, USA). An unpaired t-test, one-way ANOVA, and Pearson correlation coefficient analysis were carried out to analyze significant differences and relationships. Data were represented as the mean ± SEM. *p* < 0.05 was considered statistically significant.

## 3. Results

### 3.1. Preparation and Characterization of PFC@O_2_

**Figure 1:**
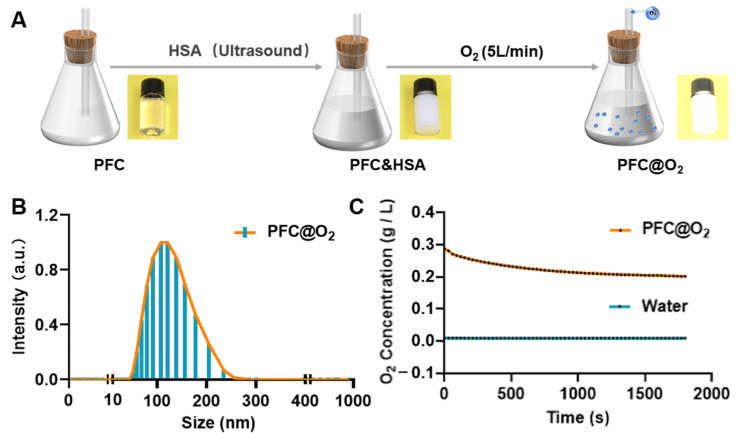
The preparation and characterization of PFC@O_2_. (**A**) Schematic diagram of the preparation of PFC@O_2_. (**B**) Hydrodynamic size of PFC@O_2_ measured by DLS. (**C**) The oxygen carrying capacity of PFC@O_2_ was measured by a JPBJ-608 portable dissolved oxygen meter.

The preparation of PFC@O_2_ was performed as shown in Figure 1A. Coating HSA on the surface of a water-insoluble PFC emulsion with ultrasound increased its solubility. This was followed by O_2_ loaded into PFC and HSA for 1 min at a flow rate of 5 L/min. The average particle size of PFC@O_2_ was 116 nm (Figure 1B). Its oxygen carrying capacity was verified in vitro, the PFC@O_2_ could carry 25 times the concentration of oxygen when compared to water (Figure 1C).

### 3.2. PFC@O_2_ Inhibited HIF-1a Expression and M2-Like Macrophage Polarization In Vitro

**Figure 2:**
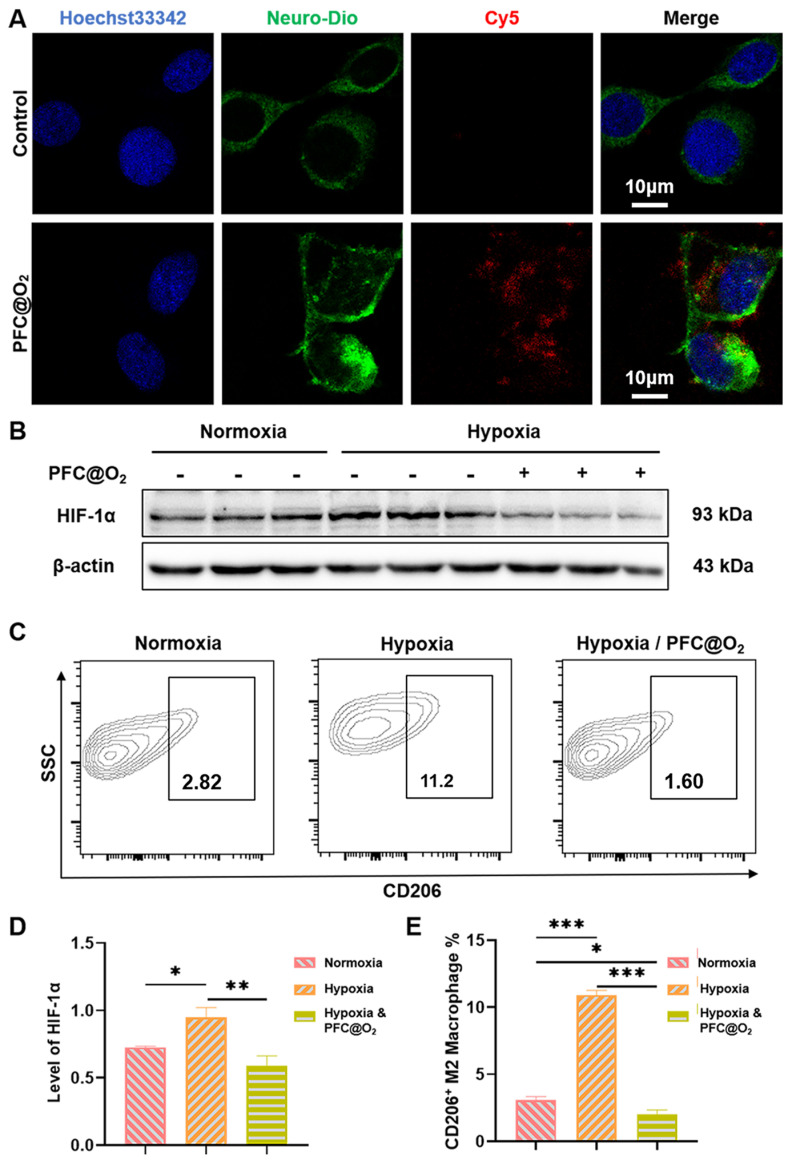
PFC@O_2_ inhibited HIF-1a expression and M2 polarization in vitro. (**A**) Confocal images of SCC7 cultured without or with PFC@O2 labeled with Cy5 for 6 h. (Blue: Hoechst 33342, Green: Neuro-DiO, and Red: Cy5). Scale bar = 10 μm. (**B**) Western blotting revealed the expression of HIF-1α in SCC7 cells cultured in normoxia, hypoxia, and hypoxia conditions with PFC@O2. (**C**) Representative images of the ratio of CD206^+^ M2-like macrophage when RAW264.7 was co-cultured with SCC7 under normoxia, hypoxia, and hypoxia conditions with PFC@O2. (**D**,**E**) Quantitative analysis of (**B**,**C**) used one-way ANOVA (n = 3 per group). (* *p* < 0.05, ** *p* < 0.01, and *** *p* < 0.001).

Co-location analysis by confocal laser microscopy was used to verify whether SCC7 could uptake the PFC@O_2_. Surprisingly, Cy5-labeled PFC@O_2_ could be effectively ingested by SCC7 (Figure 2A). After that, Western blotting showed that the expression of HIF-1α in the PFC@O_2_ treatment group was significantly decreased, while in the hypoxia group, the expression was significantly upregulated (Figure 2B). Quantitative analysis showed significant statistical differences (Figure 2C). Flow cytometry was used to detect the proportion of CD206^+^ M2-like macrophages. SCC7 and RAW264.7 were co-cultured under normoxia, hypoxia, and hypoxia with PFC@O_2_ treatment. Compared with the hypoxia and normoxia groups, PFC@O_2_ could significantly inhibit the polarization of M2-like macrophages (Figure 2C,E).

### 3.3. PFC@O_2_ Inhibited 4NQO-Induced OSCC In Situ by Downregulation of the Expression of HIF-1α

**Figure 3:**
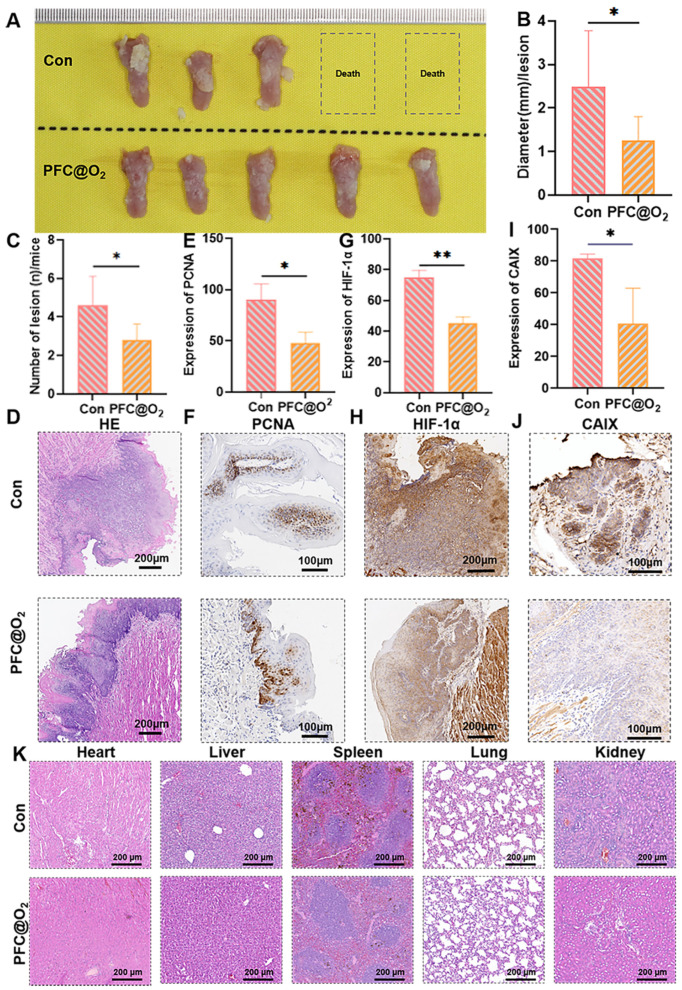
PFC@O_2_ inhibited the progression of OSCC in situ by downregulation of the expression of HIF-1α. (**A**) Image of tumor from 4NQO-induced OSCC in both the control and PFC@O_2_ groups. Quantitative analysis of tumor (**B**) size and (**C**) number of lesions in the control and PFC@O_2_ groups. (* *p* < 0.05, ** *p* < 0.01) (**D**) Representative HE stain images of the control and PFC@O_2_ groups. (**E**,**F**). Representative image and Quantitative analysis of PCNA in control and PFC@O_2_ groups (*n* = 3 per group). (**G**,**H**) Representative image and quantitative analysis of HIF-1α in the control and PFC@O_2_ groups (*n* = 3 per group). (**I**,**J**). Representative image and quantitative analysis of CA IX in the control and PFC@O_2_ groups (*n* = 3 per group). (**K**) Representative HE stain images of the heart, the liver, the spleen, the lung, and the kidney in the control and PFC@O_2_ groups.

Compared with the untreated control group, the PFC@O_2_ treatment group had significantly reduced tumor volumes and numbers (Figure 3A–C), tumor tissue HE is shown in Figure 3D, and IHC showed that the expression of PCNA in the PFC@O_2_ treatment group was significantly reduced (Figure 3E,F), which indicated that PFC@O_2_ could significantly inhibit the growth of OSCC. The expression of HIF-1α (Figure 3G,H) and its target genes-carbonixc anhydrase 9 (CA IX) (Figure 3I,J) in the treated group was also significantly lower than that of the control group, indicating that PFC@O_2_ treatment could reverse the hypoxic TME and inhibit the progression of OSCC by targeting HIF-1α. At the same time, a HE stain of the heart, the liver, the spleen, the lung, and the kidney in both groups showed no obvious abnormalities, indicating that PFC@O_2_ treatment had good biocompatibility (Figure 3K).

### 3.4. PFC@O_2_ Reversed the Immunosuppressed TME

**Figure 4:**
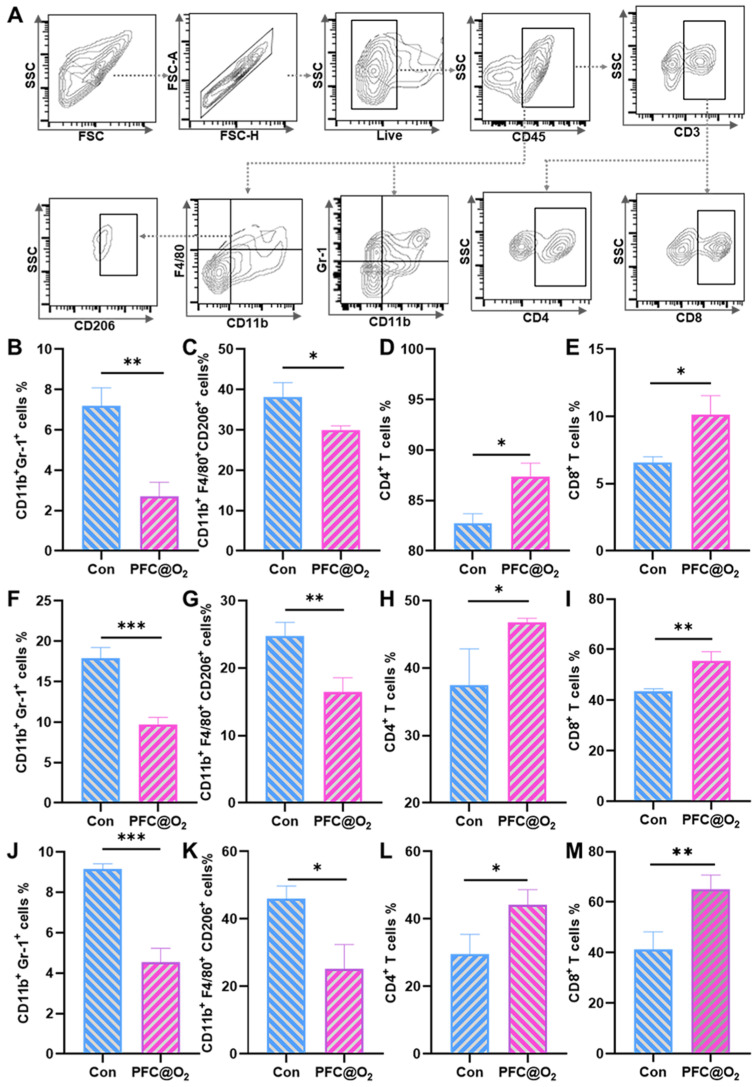
PFC@O_2_ reversed the immunosuppressed TME. (**A**) A schematic gating strategy of immune cells in a 4NQO-induced mouse OSCC model. Flow cytometric quantification of (**B**–**E**): the ratio of CD11b^+^ Gr-1^+^ MDSCs, CD11b^+^ F4/80^+^ CD206^+^ M2-like macrophages, CD4^+^, and CD8^+^ T cells in the OSCC TME of mice. (**F**–**I**) The ratio of CD11b^+^ Gr-1^+^ MDSCs, CD11b^+^ F4/80^+^ CD206^+^ M2-like macrophages, CD4+, and CD8+ T cells in the spleen of mice. (**J**–**M**) The ratio of CD11b^+^ Gr-1^+^ MDSCs, CD11b^+^ F4/80^+^ CD206^+^ M2-like macrophages, CD4^+^, and CD8^+^ T cells in the lymph nodes of mice. *n* = 3 per group. (* *p* < 0.05, ** *p* < 0.01, and *** *p* < 0.001).

It has been found that abundant M2-like tumor-associated macrophages (TAMs) and MDSCs are two key factors of TIME and intratumoral delivery of O_2_. NO can effectively reverse TIME and enhance anti-tumor immunity [29]. Therefore, flow cytometry analysis was used to detect the infiltration of immune cells into tumor tissues. A schematic gating strategy for immune cells in a 4NQO-induced OSCC mouse model is shown in Figure 4A. Compared with the control group, the proportion of MDSCs and M2 macrophages in the PFC@O_2_ treatment group was significantly reduced (Figure 4B,C), and the proportion of CD4^+^ and CD8^+^T cells with anti-tumor effects was significantly increased by flow cytometric quantification analysis (Figure 4D,E). The same trend can be observed in both the spleen (Figure 4F–I) and lymph nodes (Figure 4J–M). These results suggest that PFC@O_2_ could improve hypoxia and reduce the immunosuppressive effects of MDSC and M2-like macrophages, thus enhancing the adaptive immune response to exert anti-tumor effects.

### 3.5. The Expression of HIF-1α and CA IX Were Positively Correlated with the Ratio of CD68^+^/CD163^+^ Macrophages Infiltrated in Human OSCC

**Figure 5:**
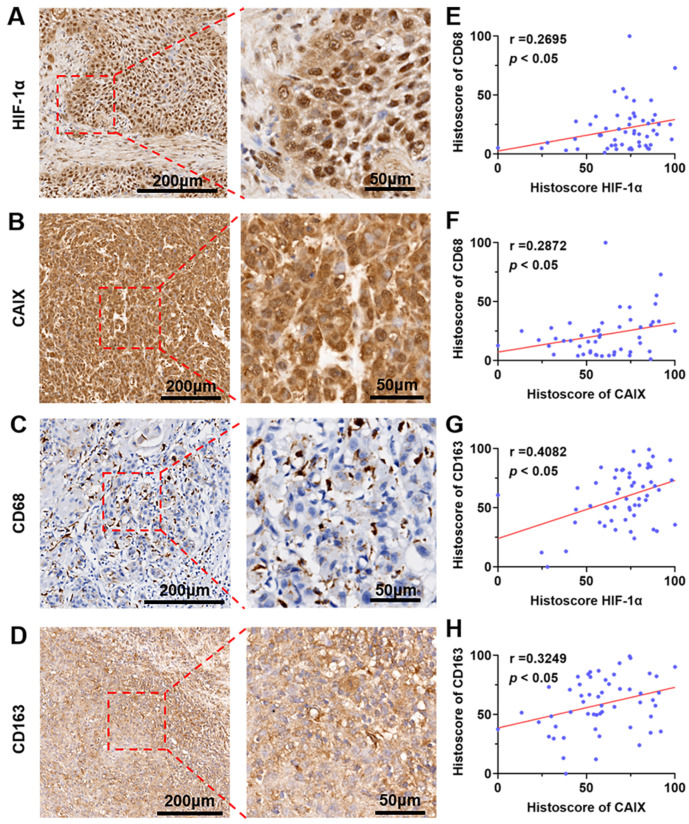
Hypoxia-relative gene expression was positively correlated with CD68^+^/CD163^+^ TAM in human OSCC. Representative images of (**A**) HIF-1α, (**B**) CAIX, (**C**) CD68, and (**D**) CD163 expression in human OSCC. The correlation analysis of (**E**) HIF-1α and CD68, (**F**) CAIX and CD68, (**G**) HIF-1α and CD163, and (**H**) CAIX and CD163 in human OSCC (*n* = 55).

Based on the findings of the above in vitro and animal experiments, we collected 55 clinical samples of OSCC after obtaining the approval of the Medical Ethics Committee of the Stomatological Hospital of Southern Medical University and the informed consent of OSCC patients. Immunohistochemistry showed that HIF-1α/CA IX, as well as CD68^+^ / CD163^+^ Macrophages were generally highly expressed (Figure 5A–D). Meanwhile, correlation analysis showed that HIF-1α/CA IX were positively correlated with the infiltration of CD68^+^/CD163^+^ macrophages in OSCC patients (Figure 5E–H). These results indicated that hypoxia and M2-like macrophages mediated immunosuppression and were closely related to the occurrence and development of OSCC.

## 4. Discussion

The multimodal treatment methods for OSCC include surgical resection, adjuvant radiotherapy and chemotherapy, immunotherapy, targeted therapy, etc. [3]. However, the 5 years survival rate of OSCC is approximately 60%, and what accounts for its low response rate to therapy is the hypoxic TME [4,30]. In view of this, nanomaterials with an oxygen storage function, such as PFC and Hb, have attracted considerable attention from scientists [31]. A growing body of research has demonstrated the efficacy and feasibility of targeting hypoxic TME and combining it with other cancer therapies [18,22,24,25]. In our study, PFC@O_2_, whose average particle size was 116 nm, carried a large amount of oxygen and slowly released it to the tumor site through blood circulation. According to article [20], approximately 25 times as much oxygen could be slowly released into TME as compared to water to relieve hypoxia. At the same time, HE staining of the heart, liver, spleen, lung, and kidney of mice showed that PFC@O2 was greatly biocompatible.

As a key regulator of tumor hypoxia, HIF-1α is commonly highly expressed in solid tumors and is closely related to treatment failure and poor prognosis [7,11,12]. Similarly, the overexpression of HIF-1α was found in OSCC tissue specimens by IHC staining in our experiment. We also found that PFC@O_2_ could significantly downregulate HIF-1α expression compared with the hypoxia group in an OSCC cell line. Additionally, in an 4NQO-induced OSCC in situ mouse model, PFC@O_2_ significantly inhibited the tumor number and size as well as weakening the expression of the proliferative marker PCNA, which may be related to the improvement of hypoxia and downregulation of HIF-1α expression by PFC@O_2_. Taking the short half-life of HIF-1α into consideration, IHC staining of CA IX, which is a target gene of HIF-1, was examined to reveal the hypoxic condition. Consistent with other research [32], our results revealed that CA IX was also highly expressed in OSCC tissue specimens. However, accumulated evidence also demonstrated that HIF-1α could be stabilized and detected under specific circumstances, e.g., nomorxia or pseudohypoxia [10,33]. As shown by our results in Figure 2B, compared with hypoxia, HIF-1α could also be detected in normoxia; however, the specific mechanism needs to be further explored.

A large number of studies have shown that HIF-1α is closely related to immunosuppressive immune cells in TME, such as MDSCs and TAMs [12]. HIF-1α can effectively recruit MDSCs and M2-like macrophages by interacting with TME chemokines such as CCL26, CCL2, and CCL5 [16,17]. According to the accumulated evidence, we know that advanced solid tumors exhibit hypoxic areas within infiltrated tumors associated with macrophages (TAMs) similar to M2 macrophages [14]. Some hypoxia-dependent signaling pathways, e.g., semaphorin3A/neuropilin-1, may promote TAMs’ migration toward the hypoxic area [34]. Thereafter, TAMs may generate a positive feedback loop through hypoxia-induced gene expression, e.g., increased CXCR4 and CXCL12 and suppressed CCR2 and CCR5 expression on macrophages by HIF-1α, to further elicit and accumulate TAMs in hypoxic areas [14,35]. Given that HIF-1α is of great importance in TAMs accumulating, the strategy of targeting MDSCs and M2-like macrophages through HIF-1α may be an effective method to inhibit tumor progression [36,37]. Furthermore, we also verified that PFC@O_2_ could effectively inhibit the differentiation of RAW264.7 macrophages into M2-like macrophages in vitro. Meanwhile, flow cytometry analysis showed that the proportion of MDSCs and M2-like macrophages in TME was also significantly reduced, and the infiltration of CD4^+^ and CD8^+^T cells was increased, which exert an anti-tumor effect. The same trend was also observed in the spleen and lymph nodes of mice. Furthermore, the relationship between HIF-1α/CA IX and the CD68^+^/CD163^+^ macrophage was verified in a human tissue specimen. CD68, CD163, and CD206 are used as markers to label M2 TAMs [38]. Taken together, these results demonstrate that PFC@O_2_ treatment can alleviate tumor immunosuppression by downregulating the expression of HIF-1α and reducing the proportion of negative immune regulatory cells. However, the relationship between HIF-1α and the recruitment and function of the immune cell population needs to be explored further.

In summary, our experiment demonstrated the feasibility of this strategy, which merely improved the hypoxic TME, although there was no synergy with other therapeutic approaches. Accumulating evidence has shown that targeting tumor hypoxia combined with immunotherapy, photodynamic therapy, and sonodynamic therapy can effectively treat tumors. In general, targeting the hypoxic TME is a promising strategy for cancer treatment. PFC, acting as a highly efficient oxygen carrier, can be used as an adjuvant for tumor therapy or synergistic tumor therapy.

## Data Availability

Not applicable.
